# Exploring effects of anesthesia on complexity, differentiation, and integrated information in rat EEG

**DOI:** 10.1093/nc/niae021

**Published:** 2024-05-16

**Authors:** André Sevenius Nilsen, Alessandro Arena, Johan F Storm

**Affiliations:** Department of Molecular Medicine, Institute of Basic Medical Sciences, University of Oslo, Sognsvannsveien 9, Oslo 0372, Norway; Department of Molecular Medicine, Institute of Basic Medical Sciences, University of Oslo, Sognsvannsveien 9, Oslo 0372, Norway; Department of Molecular Medicine, Institute of Basic Medical Sciences, University of Oslo, Sognsvannsveien 9, Oslo 0372, Norway

**Keywords:** anesthesia, consciousness, eeg, rat, complexity, integrated information, perturbational complexity index

## Abstract

To investigate mechanisms underlying loss of consciousness, it is important to extend methods established in humans to rodents as well. Perturbational complexity index (PCI) is a promising metric of “capacity for consciousness” and is based on a perturbational approach that allows inferring a system’s capacity for causal integration and differentiation of information. These properties have been proposed as necessary for conscious systems. Measures based on spontaneous electroencephalography recordings, however, may be more practical for certain clinical purposes and may better reflect ongoing dynamics. Here, we compare PCI (using electrical stimulation for perturbing cortical activity) to several spontaneous electroencephalography-based measures of signal diversity and integrated information in rats undergoing propofol, sevoflurane, and ketamine anesthesia. We find that, along with PCI, the spontaneous electroencephalography-based measures, Lempel–Ziv complexity (LZ) and geometric integrated information (Φ^**G**^), were best able to distinguish between awake and propofol and sevoflurane anesthesia. However, PCI was anti-correlated with spontaneous measures of integrated information, which generally increased during propofol and sevoflurane anesthesia, contrary to expectations. Together with an observed divergence in network properties estimated from directed functional connectivity (current results) and effective connectivity (earlier results), the perturbation-based results seem to suggest that anesthesia disrupts global cortico-cortical information transfer, whereas spontaneous activity suggests the opposite. We speculate that these seemingly diverging results may be because of suppressed encoding specificity of information or driving subcortical projections from, e.g., the thalamus. We conclude that certain perturbation-based measures (PCI) and spontaneous measures (LZ and Φ^**G**^) may be complementary and mutually informative when studying altered states of consciousness.

## Introduction

The question of how consciousness arises from brain activity has been termed the “biggest unsolved mystery in biology” (Koch 2004, Michel et al. 2019) and has been hotly debated and investigated in the last decades (Crick and Koch 1990, Searle 1998, Koch 2004). One important empirical approach is to compare physiological and psychological conditions assumed to differ in terms of state or level of consciousness, such as sleep, dreaming, anesthesia, and disorder of consciousness. Numerous methods for distinguishing such conditions from wakefulness have been proposed (see, e.g., Schnakers et al. 2013, Mashour and Hudetz 2018, Nilsen et al. 2020, Sarasso et al. 2021). However, to investigate underlying neural mechanisms of different brain states, it is essential to use animal experiments, which facilitate a range of more powerful, invasive techniques that can rarely be applied to humans and only when clinically necessary. For example, intracranial electroencephalography (EEG)/electrocorticography substantially improves the signal-to-noise ratio compared to scalp EEG (Parvizi and Kastner 2018, Tam et al. 2019) and eliminates contamination from muscle activity, which is known to affect widely used measures of anesthetic depth such as the bispectral index (Bruhn et al. 2000, Lu et al. 2008). Animal models also allow high-resolution methods such as silicon probes with high-density electrode arrays (e.g. neuropixels; Steinmetz et al. 2021) and *in vivo* whole-cell patch-clamp recordings (Noguchi et al. 2021), which can further elucidate mechanisms at the columnar or neural level, respectively. However, because animals cannot verbally report their conscious experiences, it is essential to extend existing proposed objective measures of consciousness to animals. Here, we aim to test and compare the behavior of different metrics in rats during wakefulness and during exposure to different anesthetics.

A particularly promising measure is the perturbational complexity index (PCI; Casali et al. 2013). PCI is inspired by the integrated information theory (IIT; Oizumi et al. 2014, Tononi et al. 2016). By using a perturbational approach, PCI is designed to assess the overall capacity for joint causal integration and differentiation of information, quantified as the electrophysiological complexity of cortical evoked responses, independently of sensory processing and behavior. In several studies, PCI has shown ∼100% accuracy in distinguishing states where subjects reported having been conscious and states where subjects provided no report or reported having been unconscious (Casali et al. 2013, Casarotto et al. 2016, Sarasso et al. 2014, but see Sevenius Nilsen et al. 2022, for a discussion on subjective reports in states of assumed unconsciousness). A new version of PCI (PCI^ST^; Comolatti et al. 2019) calculates the complexity of the neural response to perturbation in sensor space (rather than source space), which dispenses with the need for source reconstruction, allowing for generalization of the algorithm’s application. PCI^ST^ has already been extended to rodents by employing electric stimulation rather than transcranial magnetic stimulation (TMS), with similar results as observed with TMS-based PCI in humans (Arena et al. 2021, Cavelli et al. 2023).

The perturbational approach is considered essential for exploring causal interactions (cf. Pearl causality; Otsuka 2012) as opposed to a correlational approach, which may be confounded by noise or common drivers (Massimini et al. 2005, Casali et al. 2013). Thus, PCI serves to probe the capacity for integration of information in a system (Sarasso et al. 2021), which is considered a prerequisite for consciousness according to several current theories (e.g. Oizumi et al. 2014, Mashour et al. 2020). However, PCI, even in its generalized form, is a technically demanding and time-consuming method requiring a suitable stimulation and recording setup (typically TMS-EEG; Casali et al. 2013, but see also Arena et al. 2021, D’Andola et al. 2018) and multiple trials (perturbations) in order to identify the deterministic responses. This makes PCI ill-suited for detecting rapid fluctuations and long-term monitoring.

In contrast, measures based on spontaneous EEG signals can be used for live monitoring, over long time periods, without requiring neural perturbation. In addition, measures based on spontaneous EEG (henceforth dubbed “spontaneous measures”) might be better able to capture the current cortical state and its ongoing activity and content (Wainio-Theberge et al. 2021). For example, in a previous study (Farnes et al. 2020), we suggested that PCI and spontaneous signal diversity reflect distinct but complementary aspects of brain properties related to consciousness. Specifically, PCI may be primarily indicative of the brain’s fundamental, intrinsic capacity for or ‘level’ of consciousness (remaining essentially constant during wakefulness), while spontaneous signal diversity may be more indicative of some aspect of the current experience or ‘content’ of consciousness (decreasing when no visual stimuli is present such as when eyes are closed during wakefulness and increasing in certain conditions like during psychedelic experiences; Farnes et al. 2020). This distinction may be important when assessing disorder of consciousness patients as some evidence suggests that a high PCI value in such patients (i.e. high “capacity”) is promising in terms of long-term prognosis (Casarotto et al. 2016), irrespective of current dynamics.

Several spontaneous measures are also able to distinguish wakefulness from states assumed to be associated with reduction or absence of consciousness. For example, the power frequency distribution of synchronized neural activity (as measured by, e.g., power spectral density of the EEG) reliably shifts toward slower dynamics in states such as anesthesia and disorder of consciousness, relative to wakefulness (e.g. Hutt 2013, Engemann et al. 2018, Lendner et al. 2020, Colombo et al. 2023). The frequency distribution of EEG is also the basis for many anesthesia monitors (e.g. bispectral index; Rampil 1998), and spectral properties like the slope of the power frequency relationship (spectral exponent) have been shown to correlate with PCI in both humans (Colombo et al. 2019) and rats (Arena et al. 2021). The link between PCI and the frequency distribution of synchronized neural activity is suggested to depend on the overall excitation/inhibition balance of the brain (Gao et al. 2017), which is important for propagation of information (Poil et al. 2012), and GABAergic inhibition has been shown to modulate cortical complexity as measured by PCI (Barbero-Castillo et al. 2021).

Measures that aim to capture more directly the fundamental properties captured by PCI (i.e. information differentiation and integration) have also shown promise in separating various states in humans (Schartner et al. 2015, 2017, Kim et al. 2018, Kim and Lee 2019, Farnes et al. 2020) and animals (Afrasiabi et al. 2021, Abásolo et al. 2015, Hudetz et al. 2015, Leung et al. 2021). However, such measures have yet to be compared directly with PCI in rodents using multiple anesthetic agents. If a strong correlation between spontaneous and perturbed measures of differentiation and integration can be found, this may expand the repertoire of available measures that can be used for empirical studies of consciousness, better assess the findings based on individual measures, and help explain when and why perturbational complexity changes during altered states of consciousness.

In the current study we aim to (i) measure spontaneous signal diversity, spectral slope, and estimates of integrated information, in rats undergoing three different kinds of anesthesia, and (ii) test the behavior of these measures relative to PCI during wakefulness and anesthesia.

## Materials and Methods

### Setup

**Figure 1.**
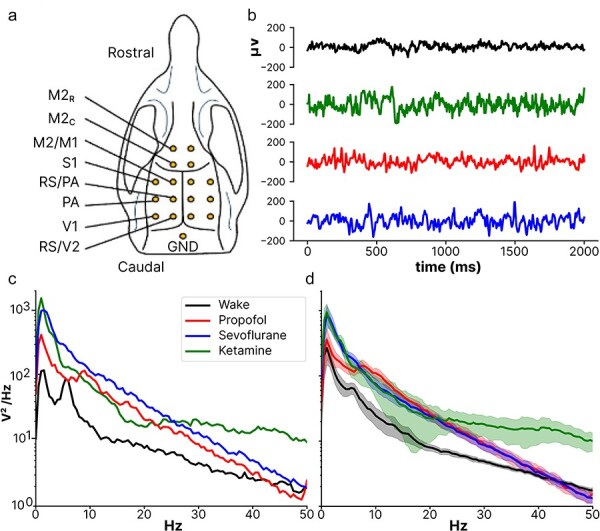
a) Overview of electrode placement and coordinates from bregma. b) Example epoch of raw data from one channel (V1, right). c) Power spectral density corresponding to (B) calculated using Welch’s method. d) Average power spectral density across trials and rats with two standard errors shaded.

Detailed methods can be found in Arena et al. (2021). Briefly, adult male Sprague-Dawley rats (300–550 g, *n* = 12) were implanted with 16 chronic epidural stainless steel screw electrodes (1.2 mm caliber; Centrostyle, Vedano Olona, Italy). The screws were in direct contact with the dura by penetration of the skull and placed along a symmetric grid along the sagittal suture (Fig. 1a). Average impedances for all electrodes (measured *in situ* with 1 kHz) at the beginning of each recording session was 7.12 ± 0.42 kΩ.

One week after surgery, recovery, and habituation to body and head restriction stands, rats underwent electrophysiological recording sessions consisting of a ∼45 min acclimatization period, a period of normal rest, followed by one of three different anesthetics (ketamine: 30 mg/kg/min induction and 1.75 mg/kg/min maintenance, propofol: 10 mg/kg/min and 1 mg/kg/min, and sevoflurane: 5% and 2.5% end-tidal concentration). Every third minute, responsiveness was investigated by pinching the tail with a forceps. Mean maintenance dosages across rats and sessions were sevoflurane: 2.58 ± 0.03%, propofol: 1.06 ± 0.02 mg/kg/min, and ketamine: 1.83 ± 0.03 mg/kg/min. Periods of burst suppression, as evidenced by EEG, were excluded. Seven rats were exposed to all three different anesthetics, and one rat was exposed to propofol and sevoflurane anesthesia. The order of anesthetics was randomized for each rat with a rest period in between (>48 h).

Each recording consisted of 80–100 pulses of electrical monophasic stimulation (1 ms duration, 0.1 Hz, 50 μA) that produced evoked potentials suitable for calculation of PCI^ST^. Raw epidural recordings were visually inspected for bad channels (numerous artifacts, amplitudes >1 MΩ, not reflecting neural activity), then all channels were re-referenced to a bipolar frontal-occipital derivation (M2-V2 right). EEG was then down-sampled to 500 Hz, and bandpass filtered between 0.5 and 45 Hz (Butterworth filter, fourth order), before epoched into 4 s non-overlapping epochs time locked prior (−5000 to −1000 ms) to the electrical monophasic pulse stimulation. To ensure an equal basis for analysis, 14 channels and 80 epochs were randomly selected for each rat in each condition, after removal of some channels and epochs due to artifacts or noise.

### Analysis

Analysis followed two steps: (1) replicate previous results of alterations in signal diversity and spectral properties observed in humans and (2) compare PCI^ST^ with measures of signal diversity and information integration. Follow up *post hoc* tests were formulated after initial analysis to better understand the relationships between the observed results: (3a) relationship between observed results and estimated network properties based on functional connectivity, and (3b) reproduction of relationship between measures of integrated information, signal diversity, and network properties, in an auto-regressive model. See Table 1 for a brief overview of measures used and Appendix 1 for a short description, with mathematical formalism in Supplementary Table A1.

**Table 1.** Overview of measures

Name		Reference
Perturbational complexity index	PCI^ST^	(Comolatti et al. 2019)
Lempel–Ziv complexity	LZs	(Halder et al. 2021; Schartner et al. 2015)
Amplitude coalition entropy	ACE	(Schartner et al. 2015)
Synchrony coalition entropy	SCE	(Schartner et al. 2015)
Spectral exponent in the 20–40 Hz range	SE_20-40_	(Colombo et al. 2019)
Geometric integrated information	**Φ** ^G^	(Oizumi et al. 2016b)
Stochastic interaction	SI	(Barrett et al. 2011)
Decoder-based Integrated Information	Φ*	(Oizumi et al. 2016a)
Mutual integrated information	MII	(Barrett et al. 2011)
Multi-mutual information given covariance	MI	(Ay 2015)
Mean coherence (absolute pairwise correlation)	C	
Global efficiency (mean inverse path length)	GE	(Dijkstra 1959; Latora and Marchiori 2001)
Modularity for directed weighted networks	Q	(Dugué and Perez 2015)
Mean connection strength	μW_ij_	
Directed transfer function	DTF	(Kamiński and Blinowska 1991)

See Appendix 1 and Supplementary Table A1 for more details.

#### (1) Replicate previous results from humans

To investigate whether alterations in measures of signal diversity observed in humans undergoing anesthesia can be reproduced in rodents, we calculated Lempel–Ziv complexity (LZ), amplitude coalition entropy (ACE), and synchrony coalition entropy (SCE) (Schartner et al. 2015). In addition, we also aimed to reproduce earlier reported changes in spectral slope (Colombo et al. 2019, Lendner et al. 2020). LZ was calculated on a single-channel basis (LZs) and then averaged (as in Halder et al. 2021) and on a multichannel basis (LZc) as per (Schartner et al. 2015). Only single-channel results (LZs) are presented here as a complement to the multichannel ACE measure (results did not differ between the two LZ variants).

#### (2) Compare PCI^ST^ to measures of signal diversity and integrated information

Since PCI is supposed to reflect a system’s level of joint causal integration and differentiation (Casali et al. 2013), we correlated PCI^ST^ (from Arena et al. 2021) with values from the above spontaneous measures of signal diversity (as measures of differentiation) and a selection of proposed measures of integrated information developed as pragmatic alternatives to the theoretical measure used in IIT: decoder-based integrated information (Φ*; Oizumi et al. 2016a), geometric integrated information (**Φ**^G^; Oizumi et al. 2016b), stochastic interaction (SI; Barrett et al. 2011), multi-mutual information given covariance (MI; Ay 2015), and mutual integrated information (MII; Barrett et al. 2011). These measures all consider what is lost by cutting the whole system into parts and differ in how these cuts are performed and how the difference is calculated.

All comparisons with PCI^ST^ were performed using Spearman’s rank order correlation, and contrasts between conditions were done pairwise with the Wilcoxon signed-rank test. Spectral exponent was calculated using custom scripts in Python 3.6, measures of signal diversity was calculated with the PyConscious package (https://github.com/andresni/pyconscious), while measures of integrated information were calculated using Matlab (R2020b) with Phitoolbox (https://github.com/oizumi-lab/PhiToolbox) (Kitazono and Oizumi 2018). The calculation of the integrated information measures employed a Gaussian approximation of the state space (rather than discrete), the Queyranne algorithm for the minimum information partition search (rather than full search), and a hierarchical partitioning for complex search algorithm (rather than exhaustive). For the definition of ‘complex’ and ‘minimum information partition’, see Oizumi et al. (2014).

#### (3a) Post hoc analysis of network topology

The first *post hoc* analysis focused on whether altered functional connectivity/network topology could explain the observed results, as an association between measures of integrated information and characteristics of network topology (e.g. network modularity, connectivity degree, and density) has been observed previously (Kim et al. 2018, Mediano et al. 2019, Toker et al. 2019). Specifically, we investigated how anesthesia influenced estimates of network integration and segregation based on functional connectivity and how these properties were associated with LZs and **Φ**^G^. In addition, as correlated noise may influence results (Mediano et al. 2019), we calculated mean coherence across channels as an estimate of external noise influencing the system.

To estimate topological features in the experimental data, we first calculated functional directed connectivity between all channel pairs by using directed transfer function (DTF) with model Order 4 (based on the optimal order estimation). While different measures of functional connectivity give diverging results for many systems (Winterhalder et al. 2005, Bakhshayesh et al. 2019), DTF was chosen as it estimates directed connectivity with edge values that are interpretable as weights, is not influenced by zero-lag effects like volume conduction (Kaminski and Blinowska 2014), and ranks highly among measures when compared on EEG-like multivariate models (Models S2B and 3 in Bakhshayesh et al. 2019). The resulting connectivity matrices were averaged across the frequency range by taking the area under the curve from 0 to 40 Hz. The estimated networks were analyzed with respect to network integration [global efficiency (GE); i.e. the mean inverse shortest path length] and network segregation (modularity; i.e. clusterization). Shortest path length was calculated with the Dijkstra algorithm (Dijkstra 1959) with edge distances as the complement of edge weights (i.e. high weight equals short distance) (see also Latora and Marchiori 2001), and modularity was calculated with the Louvain method (Blondel et al. 2008) adjusted for weighted and directed networks (Dugué and Perez 2015). Analysis was performed in Python 3.6, with bctpy for the Louvain method (https://github.com/aestrivex/bctpy, v0.5.2), pdc_dtf for DTF (https://gist.github.com/RobColeman/9bebbdbcb59d4945538e, v1.1.3), and NetworkX for the Dijkstra algorithm (https://github.com/networkx/networkx, v2.5).

#### (3b) Post hoc replication of results using an auto-regressive model

The second *post hoc* analysis aimed to test whether the relationship between measures observed in Analyses 1, 2, and 3a could be replicated in a simple auto-regressive model. This was done to investigate whether the observed results could be spurious (i.e. independent behavior of various measures) and to probe certain systemic factors that may explain the observed data.

**Figure 2.**
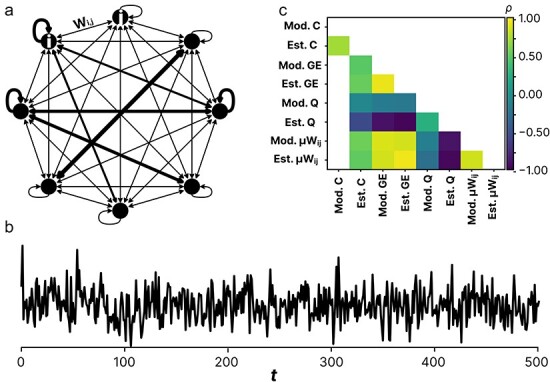
a) Abstract schematic of the auto-regressive model. b) An example time-series generated by the model. c) Spearman’s correlation (*ρ*) between network properties on the actual model parameters/network (Mod.) and estimated (Est.) from the generated data. Estimated systematic noise (Est. C) was calculated based on mean pairwise coherence of the generated data. Estimates of global efficiency (Est. GE) measuring integration, modularity (Est. Q) measuring segregation, and mean connectivity strength (Est. μWij), was calculated on connectivity matrices generated by the directed transfer function (DTF) applied to the generated data. Mod. GE., Mod Q, and Mod μWij, were calculated on the auto-regressive connectivity matrices themselves. See Appendix 1 and Table A1 for description of each measure.

We generated auto-regressive models inspired by Mediano et al. (2019) who used similar models to investigate the behavior of various measures of integrated information in relation to network characteristics. Here, each model (Fig. 2a) consisted of an *n* = 8 node network instantiated as a *n*-by-*n* matrix *A* of weights *w*, which when multiplied by each node’s current state at *t* gives the next state at *t + *1. Connection weights *w*(*i, j*) were selected from a beta distribution with *ɑ* and *β* drawn randomly from the uniform distribution [0.001, 10.1) for each network. In addition, the diagonal values (i.e. self-connections) were set to *x* times the median weight of row values (i.e. outgoing connections), with *x* drawn from the uniform distribution [3, 6]. These parameters were selected to provide networks with connectivity profiles similar to that produced by DTF which was used in estimation of functional directed connectivity in the rodent data. Finally, the auto-regressive model included additive correlated noise represented by an *n*-by-*n* matrix *ε* of noise values selected from a 0-mean multivariate normal distribution. The distribution was parameterized with a covariance matrix *c*(*i, j*), where the diagonal (standard deviation) was set to 1, and off-diagonal elements (correlation factor) set to *c*, which was drawn from a uniform distribution (0, 1). This gives the equations

-
${X_{t + 1}} = A{X_t} + {\ }{\varepsilon _t}$ ,
-
$A = {\left( {{w_{i,j}}} \right)_{1 \le i,j \le n}}$ , where $\left( {{w_{i,i}}} \right) = r*Med\left( {{w_{i,*}}} \right)$ and $r \in \left\{ {3,{\ }\ldots,6} \right\}$, and ${\left( {{w_{i,j}}} \right)_{i \ne j}} \sim Beta\left( {\alpha ,\beta } \right)$ with $\alpha ,\beta \in \left[ {0.001,{\ }\ldots,{\ }10.1} \right)$.
-
${\varepsilon _t} \sim {N_n}\left( {\mu ,\Sigma } \right)$ , where $\mu = 0$, $\Sigma = Cov\left[ {{c_i},{c_j}} \right]$, with ${c_{i,i}} = 1$, and ${\left( {{c_{i,j}}} \right)_{i \ne j}} \in \left( {0,1} \right)$.

Only matrices with a spectral radius <1 were included (i.e. ensuring stationary dynamics). A total of 1670 networks were created and simulated for *t* = 10 000 steps, producing an *n*-by-*t* time series per network. See Fig. 2b.

The time series of each network were analyzed with the same measures as applied to the recorded spontaneous rodent data (except for PCI^ST^, SCE, and spectral exponent), including estimated network properties (integration and segregation) based on DTF and mean coherence (correlated noise). To test whether DTF based on the generated time series was able to capture the underlying properties of the model, we checked the correspondence between properties estimated from the generated time-series data (noise, integration, segregation, and overall connectivity) and corresponding properties of the model. For noise, the ground truth was the correlated noise factor *c*. Estimates of network properties were strongly correlated with the ground truth (**Φ** < 0.719, *P* < .0001), except for modularity (Q) which was weakly correlated (**Φ** = 0.272, *P* < .0001). This suggests that estimating network properties based on generated data reflects the underlying ground truth. See Fig. 2c.

### Summary

Spontaneous data recorded from rats during wakefulness and anesthesia (ketamine, propofol, and sevoflurane) were used to compute a range of measures. These measures were then analyzed with respect to whether they could differentiate anesthesia versus wakefulness and cross-correlated to investigate how they behave relative to each other. Following initial analysis, we looked at how estimated network properties (integration and segregation) related to the various measures, and whether these results could be replicated in an auto-regressive model that simplistically generates dynamics similar to that of EEG.

## Results

### Measures of signal diversity, integrated information, and spectral density distinguish between wakefulness and anesthesia

**Figure 3.**
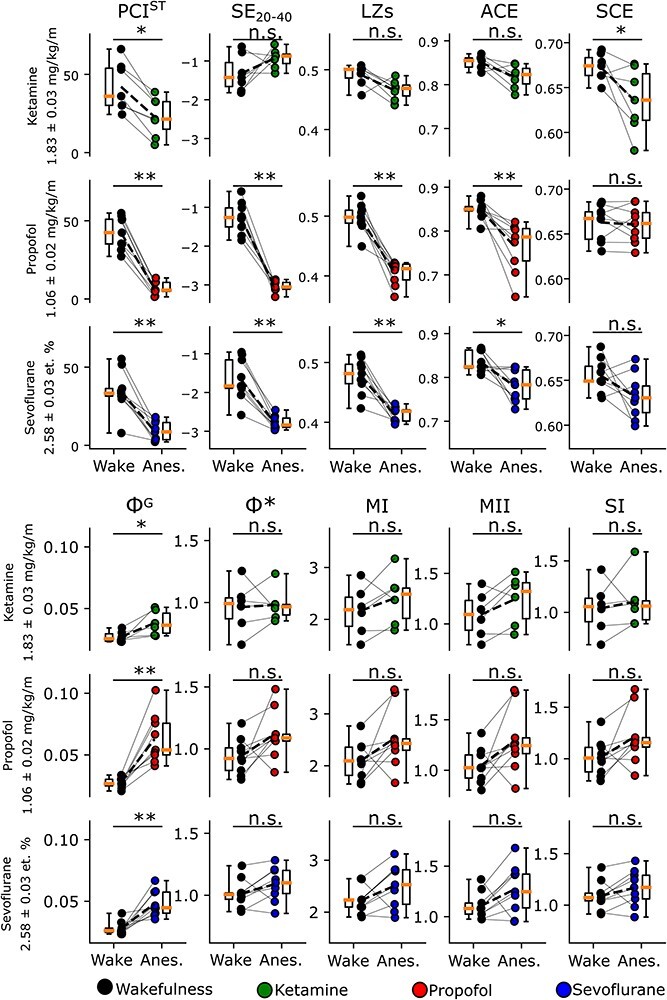
Overview of values for perturbational complexity index (PCI^ST^), spectral exponent (calculated on the 20 - 40 Hz range; SE_20-40_), Lempel Ziv complexity (LZs), amplitude coalition entropy (ACE), synchrony coalition entropy (SCE), geometric integrated information (Φ^G^), mismatched decoding based integrated information (Φ*), stochastic interaction (SI), and multi mutual information given covariance (MI), mutual integrated information (MII), et.; end-tidal. Pairwise contrasts performed with Wilcoxon S-R test from wakefulness to anesthesia, with *; *p* < .05, **; *p* < .005. Dashed line indicates the mean, with the green line indicating the median.

First, we aimed to test whether signal diversity and power spectral density are able to distinguish between wakefulness and anesthesia in rodents, as previously reported in humans. Specifically, based on previous observations, we expected a decrease in signal diversity for all anesthetics with ketamine showing the least decrease (if at all) (Ferenets et al. 2006, Casali et al. 2013, Sarasso et al. 2015, Schartner et al. 2015) and that power spectral density will be shifted toward lower frequencies (here measured by decreased spectral exponent), for all anesthetics, with ketamine showing the least shift (if at all) (Colombo et al. 2019). These observations in humans were replicated in rodents with the exception of SCE, which showed a marginal decrease during ketamine anesthesia, but no significant change for propofol or sevoflurane anesthesia. Further, measures of integrated information, while implicitly assumed to decrease during anesthesia (Oizumi et al. 2014), generally increased. However, only the measure **Φ**^G^ changed significantly relative to wakefulness. See Fig. 3 and Table 2.

**Table 2.** Mean (standard error; STE) values and Wilcoxon S-R pairwise contrasts (WSR)

	PCI^ST^	SE_20-40_	LZs	ACE	SCE
Ketamine, 1.83 ± 0.03 mg/kg/min (*n* = 7)					
Wake.	42.12 (5.96)	−1.33 (0.17)	0.49 (0.01)	0.85 (0.01)	0.67 (0.01)
Anes.	22.87 (4.77)	−0.94 (0.09)	0.47 (0.01)	0.82 (0.01)	0.64 (0.01)
WSR	*Z* = 0, *P* < .05	*Z* = 6, *P* = n.s.	*Z* = 4, *P* = n.s.	*Z* = 3, *P* = n.s.	*Z* = 2, *P* < .05
Propofol, 1.06 ± 0.02 mg/kg/min (*n* = 9)					
Wake.	42.34 (3.47)	−1.26 (0.13)	0.50 (0.01)	0.85 (0.01)	0.66 (0.01)
Anes.	6.63 (1.38)	−3.03 (0.05)	0.40 (0.01)	0.77 (0.02)	0.66 (0.01)
WSR	*Z* = 0, *P* < .005	*Z* = 0, *P* < .005	*Z* = 0, *P* < .005	*Z* = 0, *P* < .005	*Z* = 17, *P* = n.s.
Sevoflurane, 2.58 ± 0.03 et. % (*n* = 9)					
Wake.	34.66 (4.54)	−1.66 (0.18)	0.48 (0.01)	0.84 (0.01)	0.66 (0.01)
Anes.	8.98 (2.02)	−2.78 (0.06)	0.41 (0.00)	0.78 (0.01)	0.63 (0.01)
WSR	*Z* = 0, *P* < .005	*Z* = 0, *P* < .005	*Z* = 0, *P* < .005	*Z* = 3, *P* < .05	*Z* = 9, *P* = n.s.
	Φ^G^	Φ*	MI	MII	SI
Ketamine, 1.83 ± 0.03 mg/kg/min (*n* = 6)					
Wake.	0.027 (0.002)	0.963 (0.082)	2.167 (0.196)	1.090 (0.092)	1.040 (0.102)
Anes.	0.038 (0.004)	0.981 (0.055)	2.406 (0.208)	1.242 (0.102)	1.102 (0.105)
WSR	*Z* = 0, *P* < .05	*Z* = 8, *P* = n.s.	*Z* = 5, *P* = n.s.	*Z* = 3, *P* = n.s.	*Z* = 5, *P* = n.s.
Propofol, 1.06 ± 0.02 mg/kg/min (*n* = 9)					
Wake.	0.027 (0.001)	0.940 (0.049)	2.098 (0.121)	1.044 (0.057)	1.013 (0.059)
Anes.	0.063 (0.007)	1.118 (0.066)	2.527 (0.193)	1.392 (0.105)	1.210 (0.089)
WSR	*Z* = 0, *P* < .005	*Z* = 6, *P* = n.s.	*Z* = 8, *P* = n.s.	*Z* = 6, *P* = n.s.	*Z* = 7, *P* = n.s.
Sevoflurane, 2.58 ± 0.03 et. % (*n* = 9)					
Wake.	0.028 (0.002)	1.016 (0.039)	2.227 (0.089)	1.105 (0.045)	1.090 (0.048)
Anes.	0.048 (0.003)	1.088 (0.045)	2.506 (0.140)	1.269 (0.082)	1.167 (0.058)
WSR	*Z* = 0, *P* < .005	*Z* = 10, *P* = n.s.	*Z* = 10, *P* = n.s.	*Z* = 10, *P* = n.s.	*Z* = 10, *P* = n.s.

PCI^ST^, perturbational complexity index on state transitions; SE_20-40_, spectral exponent for the 20–40 Hz range; LZs; Lempel Ziv complexity, ACE; amplitude coalition entropy, SCE; synchrony coalition entropy, Φ^G^; geometric integrated information, Φ*; mismatched decoding based integrated information, SI; stochastic interaction, MI; multi mutual information given covariance, MII; mutual integrated information, n.s., non-significant; *Z*, WSR test statistic; et., end-tidal. Note that *n* = 6 for the ketamine condition for measures of integrated information due to one extreme outlier value (>10 STE).

### PCI^ST^ correlated strongly with signal diversity, spectral exponent, and integrated information

**Figure 4.**
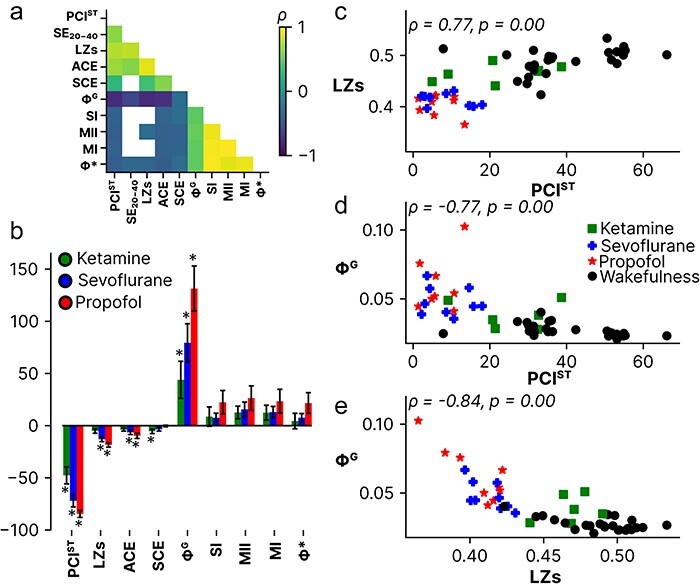
a) Spearman’s correlation (*ρ*) between measures, with non-significant correlations zeroed out (white squares). b) Relative difference, in percent, between wakefulness and anesthesia. Negative values indicate that the measure decreases during anesthesia. Stars (*) indicate a Wilcoxon S-R *p* < .05. Error bars represent one standard error of the mean. c-e) Spearman’s correlation (*ρ*) between Lempel Ziv complexity (LZs), perturbational complexity index (PCI^ST^), and geometric integrated information (Φ^G^), across conditions. Abbreviations: PCI^ST^; perturbational complexity index on state transitions, SE; spectral exponent (calculated in the 20-40 Hz range), LZs; Lempel Ziv complexity, ACE; amplitude coalition entropy, SCE; synchrony coalition entropy, Φ^G^; geometric integrated information, Φ*; mismatched decoding based integrated information, SI; stochastic interaction, MI; multi mutual information given covariance, MII; mutual integrated information.

Next, we compared the measures SE_20-40_, LZs, ACE, SCE, Φ*, **Φ**^G^, SI, MI, and MII, with PCI^ST^ to investigate whether the spontaneous EEG signal contains information that can be used for predicting PCI^ST^. Given the observed commonalities between most of these measures (except SCE) in how they change from awake to anesthesia (Fig. 3), we expected that this should be reflected in strong correlations with PCI^ST^. This expectation was supported (Fig. 4a), with PCI^ST^ being rank order correlated with all measures of signal diversity (except SCE) and integrated information (**Φ** > 0.318, *P* < .05), with the most strongly correlated measures being LZs (**Φ** = 0.767, *P* < .005), **Φ**^G^ (**Φ** = −0.762, *P < *.005), and ACE (**Φ** = 0.703, *P < *.005). SE in the 20–40 Hz frequency range was also strongly correlated with PCI^ST^ (**Φ** = 0.652, *P* < .05), as expected based on previous results (Colombo et al. 2019, Arena et al. 2021). Further, despite the low *n* for significance testing, all measures of integrated information and signal diversity (except SCE), including PCI^ST^, showed the same pattern; for all three anesthetic conditions, ketamine showed the least absolute relative change from wakefulness and propofol the largest (Fig. 4b). In other words, the relationship between the absolute magnitude of change in each condition was preserved. This seems to indicate that the general observation captures an underlying commonality despite not all pairwise contrasts passing the significance threshold. Interestingly, all measures of integrated information increased during anesthesia, while PCI^ST^ decreased. The observation of increased integrated information during anesthesia, in particular, is counterintuitive. IIT, for example, posits that integrated information should decrease in anesthesia (Oizumi et al. 2014), and practical approximations that are correlated with IIT’s proposed measure **Φ**^MAX^ (Nilsen et al. 2019) support this in animals and humans undergoing anesthesia (Afrasiabi et al. 2021, Kim and Lee 2019, Leung et al. 2021). However, the result observed here is not unprecedented, at least within specific bands of the EEG frequency spectrum (see, e.g., Kim et al. 2018). A supplementary analysis on specific frequency bands for the measures LZs and **Φ**^G^ reflected the overall results, except for lower frequencies (≤ 4 Hz). See Appendix 2 and Supplementary Fig. SA1.

### Post hoc analysis

#### Functional network integration and segregation correlate with measures of information integration and signal differentiation

Given the unexpected directional effect of anesthesia on measures of integrated information, we performed *post hoc* analyses to investigate the source of the opposite directional effects of signal diversity (decrease in anesthesia) and information integration (increase in anesthesia). See Materials and Methods.

**Figure 5.**
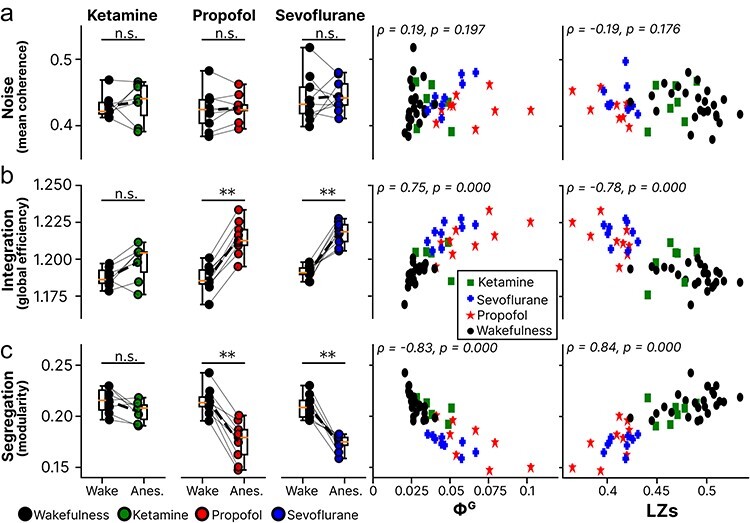
Pairwise comparisons of network properties between wakefulness and anesthesia (left), and relative to geometric integrated information (Φ^G^) (middle) and Lempel Ziv complexity (LZs) (right). a) Correlated noise, measured by average absolute spontaneous auto-correlation between all channels. b) Integration, captured by global efficiency. c) Segregation, captured by modularity. Abbreviations: n.s.; non-significant, *; *p* < .05, **; *p* < .01 (non-adjusted); *ρ*; Spearman’s correlation coefficient.

First, we investigated whether altered network topology based on the functional connectivity estimated by DTF could explain the observed results. The estimated connectivity networks were analyzed with respect to network integration and segregation. We observed that for propofol and sevoflurane anesthesia (but not ketamine), relative to wakefulness, there was a significant increase of network integration (GE) and a significant decrease of network segregation (modularity) (Fig. 5b, c left). Further, individual measurements of LZs were significantly correlated with degree of integration and segregation (**Φ** = 0.753, *P *< .0001 and **Φ** = −0.751, *P *< .0001, respectively) (Fig. 5b, c right), while **Φ**^G^ showed the inverse relationship (**Φ** = −0.437, *P *< .0001 and **Φ** = 0.4, *P *< .0001, respectively). Next, as some measures of integrated information (not **Φ**^G^) were observed to be influenced by systematic correlated noise in models previously (Mediano et al. 2019), we tested whether correlated noise (mean coherence) varied between awake and anesthesia conditions in the experimental data. We did not observe any systematic change in correlated noise between anesthesia and wakefulness (*Z* > 3.0, *P* > .078), indicating that an outside systematic factor (i.e. noise) does not explain the results (Fig. 5a right). See Table 3.

#### Opposite directional effects of signal diversity and integrated information can be reproduced in an auto-regressive model and are not due to systematic noise

**Figure 6.**
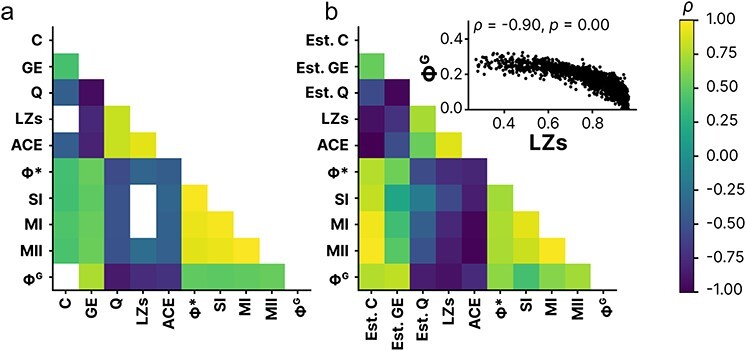
Comparison of relationship (Spearman’s correlation coefficient; *ρ*) between measures based on recorded data (a) and generated time-series of auto-regressive models (b), with the relationship between Lempel-Ziv complexity (LZs) and geometric integrated information (Φ^G^) as an inset. Non-significant correlations are zeroed out (white squares). Mean coherence is an estimate of systematic noise, global efficiency is an estimate of network integration, and modularity is an estimate of network segregation. Abbreviations: ACE; amplitude coalition entropy, SCE; synchrony coalition entropy, Φ*; mismatched decoding based integrated information, SI; stochastic interaction, MI; multi mutual information given covariance, MII; mutual integrated information.

To better understand the opposite directional effects of LZs and **Φ**^G^ in anesthesia, we generated auto-regressive models inspired by Mediano et al. (2019) in order to better probe systematic factors. First, we investigated whether the negative correlation between LZs and **Φ**^G^ in the original data was spurious by repeating the analysis on time-series data generated by the auto-regressive models. We observed that LZs and **Φ**^G^ were indeed inversely rank order correlated across model networks (**Φ** = −0.90, *P *< .0001), supporting the original finding (Fig. 6b inset).

**Table 3.** Network properties

	Ketamine 1.75 mg/kg/min			Propofol 1 mg/kg/min			Sevoflurane 2.5%		
	W	K	WSR	W	P	WSR	W	S	WSR
Mean coherence	0.430 (0.007)	0.436 (0.012)	*Z* = 11 *P* = .688	0.423 (0.010)	0.427 (0.007)	*Z* = 17 *P* = .570	0.441 (0.012)	0.445 (0.008)	*Z* = 21 *P* = .910
GE	1.188 (0.003)	1.198(0.005)	*Z* = 3 *P* = .08	1.187 (0.004)	1.214 (0.002)	*Z* = 0 *P* = .004	1.191 (0.003)	1.218 (0.003)	*Z* = 0 *P* = .004
Modularity	0.215 (0.005)	0.205 (0.004)	*Z* = 3 *P* = .08	0.215 (0.004)	0.176 (0.006)	*Z* = 0 *P* = .004	0.210 (0.004)	0.174 (0.003)	*Z* = 0 *P* = .004

Overview of mean (standard error) values for each condition: wakefulness (W), ketamine (K), propofol (P), and sevoflurane (S), with Wilcoxon S-R test (WSR) *Z*-stats and *P*-values. Values are presented for the average pairwise absolute between-channel spontaneous correlation (C, estimate of outside noise), global efficiency (GE, estimate of integration), and modularity (Q, estimate of segregation).

Secondly, we investigated the relationship between structural properties of the simulated networks and the spontaneous measures of differentiation (LZs) and integration (**Φ**^G^) (Fig. 6b). See Fig. 6a (recorded data) for comparison. The auto-regressive models showed, similar to previous studies on network topology and information integration (Kim et al. 2018, Mediano et al. 2019, Toker et al. 2019), that **Φ**^G^ was positively affected by alterations in network integration (GE: **Φ** = 0.811, *P *< .0001) and negatively by segregation (modularity: **Φ** = −0.837, *P *< .0001), with LZs being negatively affected by integration (**Φ** = −0.715, *P *< .0001) and positively affected by segregation (**Φ** = 0.739, *P *< .0001). Here, we diverge from Mediano et al. (2019), where they argued that Φ* showed different dynamics than **Φ**^G^, while they were strongly correlated in our simulated data (**Φ** = 0.659, *P *< .0001) although less so in the rodent data (**Φ** = 0.51, *P* < .001).

Next, as some measures of integrated information (not **Φ**^G^) were observed to be influenced by systematic correlated noise in models previously (Mediano et al. 2019), we aimed to reproduce the same finding in our models and test whether correlated noise varied between awake and anesthesia conditions in the experimental data. We observed that the correlated noise was weakly correlated with **Φ**^G^ across networks (**Φ** = 0.26, *P *< .0001), replicating results from Mediano et al. (2019), and negatively correlated with LZs (**Φ** = −0.43, *P *< .0001), as expected as correlated noise “decrease” functional segregation. However, estimated noise based on between-channel coherence of the generated time-series, although correlated with the noise factor in the models (**Φ** = 0.719, *P *< .0001), was strongly correlated with both LZs (**Φ** = −0.908, *P *< .0001) and **Φ**^G^ across networks (**Φ** = 0.770, *P *< .0001). See Fig. 6b.

## Discussion

### Summary

In the present study, we compared the ability of measures based on spontaneous EEG to distinguish between general anesthesia versus wakefulness in rats, relative to a leading perturbational measure of complexity, PCI^ST^.

First, we observed that propofol and sevoflurane anesthesia reduced both the spontaneous signal diversity (differentiation) and the spectral exponent (1/*f* frequency ratio in the 20–40 Hz range), whereas ketamine anesthesia produced no detectable changes in these measures. These results are in line with previous results from humans (Schartner et al. 2015, Colombo et al. 2019).

Secondly, we found that propofol and sevoflurane anesthesia increased certain spontaneous EEG-based measures of integrated information. This finding was not in line with expectations based on several previous studies (see, e.g., Afrasiabi et al. 2021, Oizumi et al. 2014, Kim and Lee 2019, Leung et al. 2021). However, our finding is not unprecedented, as Kim et al. (2018) found frequency band-limited increases in information integration in spontaneous EEG recorded in humans during propofol and ketamine anesthesia, although with a slightly different measure of integrated information than those used here. Furthermore, except for the measure SCE, all spontaneous measures implemented here showed the same overall pattern, with propofol inducing the largest absolute change and ketamine the least, relative to wakefulness, echoing PCI^ST^ values in the same animals (Arena et al. 2021). The ketamine anesthesia induces minimal or no detectable change (relative to wakefulness) in standard measures of consciousness, such as PCI and LZ, is well known (Sarasso et al. 2015, Schartner et al. 2015) and fits with the observation that a large majority of subjects undergoing ketamine anesthesia report vivid dream experiences after awakening not unlike the reports of dream experiences following rapid eye-movement sleep. Therefore, ketamine anesthesia, despite a known dose-dependent effect on cortical complexity (Li and Mashour 2019, Arena et al. 2022), is often used at low dosage as a condition of unresponsiveness combined with consciousness, unlike propofol and sevoflurane anesthesia, which is usually associated with both unresponsiveness and unconsciousness (but see Nilsen et al. 2022 for a discussion).

Finally, while all the implemented measures correlated with PCI^ST^, LZs, and **Φ**^G^ had the strongest association and were among the best measures (along with PCI^ST^ and the spectral exponent in the 20–40 Hz range) at distinguishing wakefulness from sevoflurane and propofol anesthesia. This suggests that these measures capture underlying properties of the physiological brain state important for the evoked responses that PCI is based on (such as bistable dynamics; Fecchio et al. 2019, Arena et al. 2021). Thus, although there are good reasons to use perturbational approaches such as PCI to probe the complexity of the intrinsic causal structure of the system (brain), as stressed by the IIT of consciousness (Tononi 2012, Oizumi et al. 2014, Albantakis et al. 2022), our results suggest that technically simpler measures based on spontaneous activity can be valid alternatives in some situations.

### Functional information integration and differentiation is associated with network topology

The observation that measures of integrated information increased during propofol and sevoflurane anesthesia (Fig. 3), thus being negatively correlated with PCI^ST^, seems unexpected based on predictions from IIT (Oizumi et al. 2014, Albantakis et al. 2022). Since the measures of integrated information implemented here are approximations to the more exact, but computationally intractable, measure proposed by IIT (Barrett et al. 2011, Oizumi et al. 2016a, 2016b), it is possible that these measures are not capturing (i.e. properly approximating) the kind of integrated information proposed by the theory. For example, IIT’s Phi (**Φ**^MAX^) measure requires a perturbational approach to map out the complete causal diagram of the system and finding the temporo-spatial scale that maximizes **Φ**^MAX^, neither of which are possible with PCI or other practical methods. Thus, while measures like LZ and Φ* have been shown to correlate strongly with IIT’s **Φ**^MAX^ (v3.0 of the theory; Oizumi et al. 2014) in simple model systems (Nilsen et al. 2019), it can be argued that these and similar measures, when applied to spontaneous EEG, are insufficient approximations for assessing information integration in real systems.

IIT does, however, stress that systems need to be highly integrated and differentiated to have a high **Φ**^MAX^ (Oizumi et al. 2014), and previous studies suggest that PCI is high only when systems show both characteristics (Casali et al. 2013, Sarasso et al. 2014). To further test these ideas, we investigated whether measures of differentiation (LZs) and integration (**Φ**^G^) are generally reflected in the same properties estimated from functional connectivity matrices obtained by DTF. We observed that functional integration (**Φ**^G^) correlated positively with network integration (measured by GE) and negatively with network segregation (measured by modularity). This was in line with previous studies (Kim et al. 2018, Toker et al. 2019). Signal diversity (LZs) showed the inverse relationship with network integration and segregation, relative to **Φ**^G^.

However, as DTF-based estimates of functional connectivity may not align with the ground truth, we ran an auto-regressive model approximating EEG time series and replicated the observed relationship between measures applied to the rodent data. Specifically, integrated information (**Φ**^G^) was associated positively with estimated network integration and negatively with estimated network segregation while signal diversity showed the inverse relationship. This echoed the results based on the rodent data. Furthermore, signal diversity and integrated information was negatively correlated across different auto-regressive models (as in the main results). Finally, there was a large degree of correspondence between topological properties estimated based on DTF-derived connectivity from the generated time series and the same properties calculated from the underlying connectivity of the models (Fig. 2c).

Taken together, these results support the hypothesis that both LZs and **Φ**^G^ reflect changes in the underlying topological properties observed during anesthesia in rats and that our findings here are unlikely to be spurious. However, the increased integration and reduced segregation induced by anesthesia warrant discussion.

General anesthesia is commonly considered to disrupt cerebral connectivity and integration (e.g. Bonhomme et al. 2011, Hudetz 2012, Pal et al. 2016, Chen et al. 2019), although the picture is not always so clear. For example, Paasonen et al. (2018) observed little difference in functional connectivity between wakefulness and propofol anesthesia in humans, as also reported in rats with isoflurane anesthesia (Liang et al. 2012). Liu et al. (2013) even found an increase in network integration, through a merging of the default and task executive networks, during propofol anesthesia in rats. Moreover, anesthesia is known to increase neocortical synchronization as measured by EEG (Shalbaf et al. 2015, Liang et al. 2016), and a recent review found diverse results based on different methods (Liu et al. 2021). Thus, there is still no clear consensus.

There seem to be at least two potential explanations for our observations. (i) First, anesthesia might disrupt information encoding rather than cortical integration per se (Hudetz 2006, Alkire et al. 2008, Liu et al. 2013). A loss of information encoding could result from both a decrease and increase in connectivity by reducing the specificity of information encoding. For example, visual stimulation of rats has been shown to produce a global cortical response during isoflurane and sevoflurane anesthesia (Hudetz and Imas 2007, Arena et al. 2017) but not in propofol anesthesia (Arena et al. 2017). Arena et al. (2021) also observed that PCI^ST^ depended on the laminar depth of the intracortical electrical stimulation only during wakefulness and ketamine anesthesia and not for propofol or sevoflurane anesthesia. Thus, it is possible that propofol and sevoflurane cause a loss of specificity in processing or encoding (i.e. loss of information), while the network becomes more integrated. This is also one of the proposed mechanisms causing low PCI (Sarasso et al. 2014) and might manifest as a global but homogeneous cortical response to perturbation.

However, Arena et al. (2021) showed that propofol and sevoflurane anesthesia reduced overall effective connectivity, as assessed by electrical stimulation. This is likely due to cortical bistable dynamics during anesthesia that hinder spread of activation (Sarasso et al. 2014), although stimulation at higher intensities can produce a more global response (Massimini et al. 2012). This suppression of perturbation-induced activation is also a feature of systems that self-organize into a subset of stable attractor states (Kelso 2012). A recent study in rats undergoing isoflurane anesthesia showed that a multitude of weak corticocortical connections was sufficient to constrain global activity to a limited set of states that nonetheless were globally correlated, in line with self-organization principles (Blackwood et al. 2022). Taken together, reduced overall connectivity could lead to an apparent increase in integration but only in spontaneous resting-state activity.

(ii) Our results might also be explained by preservation of thalamocortical global projections during anesthesia. In this case, even if long-range corticocortical connectivity is disrupted, the increased integration and reduced segregation, seen here in spontaneous EEG, may be due to a common thalamic driver giving the appearance of increased integration. A recent study in rats undergoing propofol anesthesia (Jiang et al. 2022) showed that corticocortical long-range connectivity was weakened, but thalamocortical projections were not. Another recent study found that isoflurane anesthesia in mice abolished systematic cortico-thalamo-cortical rebound activation resulting from cortical stimulation (Claar et al. 2023), which, according to the authors, may contribute to reduced PCI during anesthesia. While this might suggest that thalamocortical activation is suppressed, a similar study in rats undergoing isoflurane anesthesia showed that it was top-down corticothalamic pathways that were disrupted (Raz et al. 2014). In a recent paper by Hönigsperger et al. (2024), they suggested that deeper layers are relatively more important for PCI^ST^ during wakefulness in mice, and L5/L6 is known to project to the thalamus (Harris et al. 2019). Additionally, several studies have also shown that thalamic stimulation can have an arousing effect during anesthesia (e.g. Honjoh et al. 2018, Tasserie et al. 2022). While these results probably reflect effects on the ascending reticular arousal system rather than on direct ionotropic glutamatergic connectivity, it is possible that the seemingly increased integration during anesthesia observed here is orchestrated through subcortical structures.

In summary, although there is evidence for a coupling between spontaneous and evoked cortical dynamics in wakefulness (Wainio-Theberge et al. 2021), we suggest that this correlation may break down during propofol and sevoflurane anesthesia. Thus, the observed increase in spontaneous integration (reported here) and decrease in effective connectivity (Arena et al. 2021) might be due to (i) reduced information encoding specificity through cortical self-organization into a limited set of global states and/or (ii) a common subcortical driver.

One way to test these hypotheses is through investigating the effect of anesthesia combined with certain lesions. For example, a recent study showed that inter-hemispheric slow-wave propagation and symmetry was reduced during deep sleep in split-brain patients, relative to controls (Avvenuti et al. 2020). While this finding seems to support hypothesis (i) over (ii), the authors note that some residual propagation remained, possibly mediated through cortical-subcortical loops. If a similar study could be done in a callosotomy animal model with propofol or sevoflurane anesthesia, it might elucidate the origin of cortical resting-state functional integration observed in the present study.

### Limitations

Because of the rather low numbers of experimental repetitions in parts of our study, several of the seemingly contrasting results obtained in the *post hoc* analysis should be interpreted with caution, as they would not survive correction for multiple comparisons. However, given the complementing direction of effects between measures (besides SCE) and the consistency between the results from the graph theoretical analysis and the auto-regressive model, we argue that these results are suggestive and interesting, although they need corroboration.

Another limitation is the nature of the spontaneous EEG data. As each rat received electrical stimulation every 10 s (for PCI^ST^ measurements; see Arena et al. 2021), our analysis of spontaneous EEG was limited to the 4 s prior to each stimulus (to avoid lingering effects of the preceding stimulation). Therefore, our calculations of the spectral exponent, power spectral density, and integrated information had to be averaged over trials, thus preventing the use of a sliding window over time or investigating dependencies on epoch length and sampling rate. Another possible source of error is that the intermittent stimulation might disrupt the baseline activity for longer than was readily apparent.

Finally, while the measure of functional connectivity used here (DTF) is a widely used method, it is known that various measures of functional connectivity can produce wildly different results (Winterhalder et al. 2005, Bakhshayesh et al. 2019) and it is unclear which measure most accurately reflects the underlying connectivity. Especially when considering that cortical EEG is recorded further away from the neurons than LFPs. Moreover, graph theoretic measures such as GE and clustering coefficient (i.e. integration and segregation) have been shown to be dependent on the underlying connectivity measure used to generate connectivity profiles (Pullon et al. 2022). However, there was a high degree of correspondence between the graph theoretical measures based on DTF calculated on the generated time-series of the auto-regressive models and the same measures calculated on the actual connectivity matrices (Fig. 2c). While this does not validate DTF as an EEG-based measure of functional connectivity (since an auto-regressive model of EEG is overly simplistic), the overall convergence of our results between the model and the empirical data suggests that DTF is, at least, useful.

## Conclusion

We find that the information contained in the spontaneous electrophysiological data recorded at a cortical level in rats can be used to accurately distinguish between wakefulness and different anesthetic states in accordance with results from human studies and accurately match the discrimination obtained with the leading perturbation-based measure, PCI.

We also found that estimates of topological properties (GE and modularity) based on functional connectivity corresponded with alterations in measures of integration and differentiation, respectively. However, estimates of topological properties based on functional connectivity measured with spontaneous EEG diverged from those based on effective connectivity measured with perturbed EEG; anesthesia was observed to increase integration and decrease segregation, whereas a perturbational approach suggested the opposite.

We speculate that our results can be explained by a decrease in specificity in information encoding during propofol and sevoflurane anesthesia, but not in ketamine anesthesia, or that thalamocortical projections are relatively preserved during propofol and sevoflurane anesthesia, while long-range corticocortical connectivity is comparatively reduced, as suggested by previous studies. Taken together, this study provides evidence that approaches based on spontaneous and perturbation-evoked cortical electrophysiological signals are both useful in consciousness research, and suggests how these may complement each other.

## Supplementary Material

niae021_Supp

## Data Availability

Data are available at Arena et al. (2020): 10.25493/8CQN-Y8S.
