# Supplementary material for: Exploring effects of anesthesia on complexity, differentiation, and integrated information in rat EEG
Source: Neurosci Conscious. 2024 May 16;2024(1):niae021. doi: 10.1093/nc/niae021 (PMC11097907; doi:10.1093/nc/niae021)
Supplement: niae021_Supp [file niae021_supp.zip › suppl_data/Appendix_v3_final_no_figtext.docx]

## Appendix 1. Brief description of each employed measure

Following is a list of measures and their abbreviations employed in the main manuscript. See also Table A1 which shares the references used here.

**Perturbational Complexity Index (PCI) - State Transition variant (PCI^ST^)** calculates the complexity of the neural response to perturbation (e.g. transcranial magnetic stimulation or direct electrical stimulation) in sensor space (e.g. electroencephalography). This is done by first finding the singular value principal components of the multi-channel signal, splitting it in baseline (pre-perturbation) and response (post perturbation) before calculating their amplitude fluctuations over time wrt. to their lowest value. Then, for each principal component, calculate the difference in mean signal crossings of a threshold between the baseline (weighted) and evoked signal, with the threshold being that which maximizes this difference. PCI^ST^ is then the sum of the differences of all the principal components. See ([[1]](https://paperpile.com/c/VpFtYD/odmG).

**Lempel-Ziv complexity (LZs)** calculates the complexity of the recorded data. That is, each time series (each channel) is binarized based on median split of Hilbert amplitude, and then compressed with the Lempel-Ziv algorithm [[2]](https://paperpile.com/c/VpFtYD/3a7az). The compressed signal relative to a compressed shuffled dataset (i.e. randomized) constitutes LZs. See [[3]](https://paperpile.com/c/VpFtYD/QZUoq).

**Amplitude Coalition Entropy (ACE)** calculates the entropy of the global signal. That is, the binarized signal (as in LZs) across channels is concatenated time point by time point forming a series of states. The entropy of the resulting state series relative to that of a shuffled dataset constitutes ACE. See [[3]](https://paperpile.com/c/VpFtYD/QZUoq).

**Synchrony Coalition Entropy (SCE)** calculates the entropy of the global phase signal. The analysis is similar to that of ACE, except that the pairwise phase correlation rather than amplitude is used. See [[3]](https://paperpile.com/c/VpFtYD/QZUoq).

**Spectral exponent in the 20-40 Hz range (SE_20-40_)** calculates the linear slope of the power spectrum in the 20-40 Hz range. See [[4]](https://paperpile.com/c/VpFtYD/TD4KZ).

**Geometric integrated information (𝚽^G^)** calculates the integrated information of a system, over that of its parts. That is, 𝚽^G^  is the Kullback-Liebler (KL) divergence between the joint probability distribution of the states X of a system A, p(X^t^;X^t-1^), and the probability distribution of a statistically disconnected system q(X^t^;X_i_^t-1^) where *i* is a specific node in A (i.e. collapsing the joint probability distribution over ‘disconnected’ nodes). The ‘disconnection’ that produces the minimal KL difference is the statistically disconnected system. The minimized KL divergence thus captures how integrated a given system is by controlling for certain influences (connections) as if the difference is small, then the information transfer between e.g. two channels is low enough that ignoring that connection doesn’t drastically alter the probability distribution of the state space. See [[5]](https://paperpile.com/c/VpFtYD/W9Grk).

**Stochastic interaction (SI)** is similar to 𝚽^G^ except that KL is not minimized over a range of possible ‘disconnections’, but directly targeting the total fragmentation q(X_i_^t^;X_i_^t-1^), rather than q(X^t^;X_i_^t-1^). See [[6]](https://paperpile.com/c/VpFtYD/5AxzU).

**Decoder based integrated information (𝚽*)** is similar to 𝚽^G^ and SI, but calculates the mutual information *I* (rather than the KL divergence) of the joint probability distribution p(X^t^;X^t-1^) minus the mutual information of the product of the disconnected subsystems q(X_i_^t^;X_i_^t-1^). See [[7]](https://paperpile.com/c/VpFtYD/MKchT).

**Mutual integrated information (MII)** is similar to that of 𝚽*, except it subtracts the sum of mutual information over the parts, rather than the product. See [[6]](https://paperpile.com/c/VpFtYD/5AxzU).

**Multi mutual information given covariance (MI )** is similar to that of SI, except that it uses the joint distribution rather than the conditional. See [[8]](https://paperpile.com/c/VpFtYD/u4VA2).

**Mean coherence (Mean Coh. / Est. C)** is here the mean spontaneous absolute pairwise correlation between time series (ignoring frequency), and is a measure of systematic outside correlated noise (e.g. a common driver). In terms of the auto-regressive model, C (noise) is a specific parameter specifying the correlation between values drawn from a multivariate normal distribution. In effect, it’ll manifest as spontaneous correlation, e.g. as in volume conduction.

**Global efficiency (Global Eff. / GE)** is the mean inverse shortest path length of a graph, and captures how integrated a system is. See [[9]](https://paperpile.com/c/VpFtYD/lDtX) for the shortest path length definition and [[10]](https://paperpile.com/c/VpFtYD/Lu2Q) for efficiency.

**Modularity (Q)** is a clusterization measure that calculates the strength (or distance) of connections within groups, relative to those between groups. Cluster membership is initially random, before nodes are added or removed depending on their edge weights within their group and other groups. See [[11]](https://paperpile.com/c/VpFtYD/CDxk) for modularity, and [[12]](https://paperpile.com/c/VpFtYD/MOue) for modularity applied to directed and weighted graphs.

**Mean connection strength (μW_ij_)** captures the overall strength of connectivity, i.e. the absolute mean of the connectivity matrix in an auto-regressive model, or an estimate of functional connectivity based on time series data.

**Directed Transfer Function (DTF)** estimates directed functional connectivity over frequencies by calculating the influence of past values of one channel, on the present value of another channel, normalized by the influence of all other channels. See [[13]](https://paperpile.com/c/VpFtYD/1Gy3).

## Appendix 2. Measures of signal diversity and integrated information applied to specific frequency bands

Measures of integrated information in anesthesia have been observed to depend partially on frequency band [[14]](https://paperpile.com/c/VpFtYD/k0dZu). We redid the contrasts between wakefulness and anesthesia for the main measures LZs and 𝚽^G^, for three frequency bands (in addition to the original broadband): ≤4 Hz, 5-14 Hz, and ≥20 Hz. The three frequency bands were based partly on results from [[15]](https://paperpile.com/c/VpFtYD/5aGYw) which showed that the evoked responses used to calculate PCI^ST^ was differentiated among three different frequency bands, and an underlying difference in what the different frequency bands represent. Specifically, low frequencies (≤4 Hz) are suggested to reflect global synchronization of state changes, i.e. bistable dynamics [[16,17]](https://paperpile.com/c/VpFtYD/w28a6+FZTzF), the intermediate band (5-14 Hz) may reflect long-range communication dynamics [[18,19]](https://paperpile.com/c/VpFtYD/0HXSa+bnT3a), while higher frequencies (≥20 Hz) may reflect the active state, i.e. ongoing neural activation and firing [[20–22]](https://paperpile.com/c/VpFtYD/WkkAB+MkIOa+ldcCu).

Compared to the broadband results (main manuscript) in which ketamine anesthesia produced the least, if any, change relative to wakefulness, and propofol and sevoflurane producing the largest change, we observed that only the higher (≥20 Hz) and intermediate (5-14 Hz) frequency bands showed a similar pattern. Specifically, only the higher frequency band echoed the results for LZs in the broadband, while both the intermediate and higher frequency bands were similar as the 𝚽^G^ results in the broadband. While Kim et al [[14]](https://paperpile.com/c/VpFtYD/k0dZu/?noauthor=1) observed increased integrated information in all frequency bands except alpha during propofol, we observed the strongest effects for the beta and gamma bands (≥20 Hz). See Figure 5. However, Kim et al [[14]](https://paperpile.com/c/VpFtYD/k0dZu/?noauthor=1) also observed increased integrated information during ketamine anesthesia, which we did not. Note that they used a different measure of integrated information than used here, applied to EEG in humans. Further, both LZs and 𝚽^G^ in the broadband were strongly rank order correlated with themselves in the alpha band (𝜌 = .83 and .72, *p* < .01) and the beta-gamma band (𝜌 = .87 and .82, *p* < .01).

**References**

1. [Comolatti R, Pigorini A, Casarotto S, Fecchio M, Faria G, Sarasso S, et al. A fast and general method to empirically estimate the complexity of brain responses to transcranial and intracranial stimulations. Brain Stimul. 2019;12: 1280–1289. doi:](http://paperpile.com/b/VpFtYD/odmG)[10.1016/j.brs.2019.05.013](http://dx.doi.org/10.1016/j.brs.2019.05.013)

2. [Ziv J, Lempel A. A universal algorithm for sequential data compression. IEEE Trans Inf Theory. 1977;23: 337–343. doi:](http://paperpile.com/b/VpFtYD/3a7az)[10.1109/TIT.1977.1055714](http://dx.doi.org/10.1109/TIT.1977.1055714)

3. [Schartner M, Seth A, Noirhomme Q, Boly M, Bruno MA, Laureys S, et al. Complexity of multi-dimensional spontaneous EEG decreases during propofol induced general anaesthesia. PLoS One. 2015;10. doi:](http://paperpile.com/b/VpFtYD/QZUoq)[10.1371/journal.pone.0133532](http://dx.doi.org/10.1371/journal.pone.0133532)

4. [Colombo MA, Napolitani M, Boly M, Gosseries O, Casarotto S, Rosanova M, et al. The spectral exponent of the resting EEG indexes the presence of consciousness during unresponsiveness induced by propofol, xenon, and ketamine. Neuroimage. 2019;189: 631–644. doi:](http://paperpile.com/b/VpFtYD/TD4KZ)[10.1016/j.neuroimage.2019.01.024](http://dx.doi.org/10.1016/j.neuroimage.2019.01.024)

5. [Oizumi M, Tsuchiya N, Amari S-I. Unified framework for information integration based on information geometry. Proc Natl Acad Sci U S A. 2016;113: 14817–14822. doi:](http://paperpile.com/b/VpFtYD/W9Grk)[10.1073/pnas.1603583113](http://dx.doi.org/10.1073/pnas.1603583113)

6. [Barrett AB, Seth AK. Practical measures of integrated information for time-series data. PLoS Comput Biol. 2011;7: e1001052. doi:](http://paperpile.com/b/VpFtYD/5AxzU)[10.1371/journal.pcbi.1001052](http://dx.doi.org/10.1371/journal.pcbi.1001052)

7. [Oizumi M, Amari S-I, Yanagawa T, Fujii N, Tsuchiya N. Measuring Integrated Information from the Decoding Perspective. PLoS Comput Biol. 2016;12: e1004654. doi:](http://paperpile.com/b/VpFtYD/MKchT)[10.1371/journal.pcbi.1004654](http://dx.doi.org/10.1371/journal.pcbi.1004654)

8. [Ay N. Information Geometry on Complexity and Stochastic Interaction. Entropy . 2015;17: 2432–2458. doi:](http://paperpile.com/b/VpFtYD/u4VA2)[10.3390/e17042432](http://dx.doi.org/10.3390/e17042432)

9. [Dijkstra EW. A note on two problems in connexion with graphs. Numer Math. 1959;1: 269–271. doi:](http://paperpile.com/b/VpFtYD/lDtX)[10.1007/bf01386390](http://dx.doi.org/10.1007/bf01386390)

10. [Latora V, Marchiori M. Efficient behavior of small-world networks. Phys Rev Lett. 2001;87: 198701. doi:](http://paperpile.com/b/VpFtYD/Lu2Q)[10.1103/PhysRevLett.87.198701](http://dx.doi.org/10.1103/PhysRevLett.87.198701)

11. [Blondel VD, Guillaume J-L, Lambiotte R, Lefebvre E. Fast unfolding of communities in large networks. J Stat Mech. 2008;2008: P10008. doi:](http://paperpile.com/b/VpFtYD/CDxk)[10.1088/1742-5468/2008/10/P10008](http://dx.doi.org/10.1088/1742-5468/2008/10/P10008)

12. [Dugué N, Perez A. Directed Louvain : maximizing modularity in directed networks. Unpublished; 2015. doi:](http://paperpile.com/b/VpFtYD/MOue)[10.13140/RG.2.1.4497.0328](http://dx.doi.org/10.13140/RG.2.1.4497.0328)

13. [Kamiński MJ, Blinowska KJ. A new method of the description of the information flow in the brain structures. Biol Cybern. 1991;65: 203–210. doi:](http://paperpile.com/b/VpFtYD/1Gy3)[10.1007/BF00198091](http://dx.doi.org/10.1007/BF00198091)

14. [Kim H, Hudetz AG, Lee J, Mashour GA, Lee U, ReCCognition Study Group. Estimating the Integrated Information Measure Phi from High-Density Electroencephalography during States of Consciousness in Humans. Front Hum Neurosci. 2018;12: 42. doi:](http://paperpile.com/b/VpFtYD/k0dZu)[10.3389/fnhum.2018.00042](http://dx.doi.org/10.3389/fnhum.2018.00042)

15. [Arena A, Comolatti R, Thon S, Casali AG, Storm JF. General anesthesia disrupts complex cortical dynamics in response to intracranial electrical stimulation in rats. eneuro. 2021. pp. ENEURO.0343–20.2021. doi:](http://paperpile.com/b/VpFtYD/5aGYw)[10.1523/eneuro.0343-20.2021](http://dx.doi.org/10.1523/eneuro.0343-20.2021)

16. [Harmony T. The functional significance of delta oscillations in cognitive processing. Front Integr Neurosci. 2013;7: 83. doi:](http://paperpile.com/b/VpFtYD/w28a6)[10.3389/fnint.2013.00083](http://dx.doi.org/10.3389/fnint.2013.00083)

17. [Knyazev GG. EEG delta oscillations as a correlate of basic homeostatic and motivational processes. Neurosci Biobehav Rev. 2012;36: 677–695. doi:](http://paperpile.com/b/VpFtYD/FZTzF)[10.1016/j.neubiorev.2011.10.002](http://dx.doi.org/10.1016/j.neubiorev.2011.10.002)

18. [van Kerkoerle T, Self MW, Dagnino B, Gariel-Mathis M-A, Poort J, van der Togt C, et al. Alpha and gamma oscillations characterize feedback and feedforward processing in monkey visual cortex. Proc Natl Acad Sci U S A. 2014;111: 14332–14341. doi:](http://paperpile.com/b/VpFtYD/0HXSa)[10.1073/pnas.1402773111](http://dx.doi.org/10.1073/pnas.1402773111)

19. [von Stein A, Sarnthein J. Different frequencies for different scales of cortical integration: from local gamma to long range alpha/theta synchronization. Int J Psychophysiol. 2000;38: 301–313. doi:](http://paperpile.com/b/VpFtYD/bnT3a)[10.1016/s0167-8760(00)00172-0](http://dx.doi.org/10.1016/s0167-8760(00)00172-0)

20. [Steriade M, Nuñez A, Amzica F. A novel slow (< 1 Hz) oscillation of neocortical neurons in vivo: depolarizing and hyperpolarizing components. J Neurosci. 1993;13: 3252–3265. Available:](http://paperpile.com/b/VpFtYD/WkkAB) <https://www.ncbi.nlm.nih.gov/pubmed/8340806>

21. [Steriade M, Timofeev I, Grenier F. Natural waking and sleep states: a view from inside neocortical neurons. J Neurophysiol. 2001;85: 1969–1985. doi:](http://paperpile.com/b/VpFtYD/MkIOa)[10.1152/jn.2001.85.5.1969](http://dx.doi.org/10.1152/jn.2001.85.5.1969)

22. [Volgushev M, Chauvette S, Mukovski M. Precise long-range synchronization of activity and silence in neocortical neurons during slow-wave sleep. Journal of. 2006. Available:](http://paperpile.com/b/VpFtYD/ldcCu) <https://www.jneurosci.org/content/26/21/5665.short>
