# Supplementary material for: Exploring effects of anesthesia on complexity, differentiation, and integrated information in rat EEG
Source: Neurosci Conscious. 2024 May 16;2024(1):niae021. doi: 10.1093/nc/niae021 (PMC11097907; doi:10.1093/nc/niae021)
Supplement: niae021_Supp [file niae021_supp.zip › suppl_data/Table_A1.pdf]

Table A1

Name, Ref.	Symbol	Definition	Comment
Perturbational Complexity Index State Transitions [1]	PCI^{ST}	$\sum_{n=1}^{N_c} \Delta NST_n, \Delta NST_n = T_R[NST_n^{res}(\epsilon_n^*) - k \cdot NST_n^{base}(\epsilon_n^*)]$	$\epsilon_n^* = \max \Delta NST_n(\epsilon_n^*)$
Geometric Integrated Information [5]	Φ^G	$\min_{q\in\mathcal{M}_G} D_{KL}(p\,\|\,q)$	$\mathcal{M}_G$ constrained by $q(M_i^t\|X^{t-\tau}) = q(M_i^t\|M_i^{t-1}), \forall_i$
Decoder Based Integrated Information [7]	Φ^*	$I(X^{t-\tau}; X^t) - I^*(X^{t-\tau}; X^t)$	I^* uses $q(X^t\|X^{t-\tau}) = \prod_{i=1}^{m} q(M_i^t\|M_i^{t-1})$
Multi Mutual Information [8]	ϕ_{MI} / MI / ϕ_H	$\sum_{i=1}^{m} H(M_i^{t-\tau}, M_i^t) - H(X^{t-\tau}, X^t)$	
Stochastic Interaction [6]	ϕ_{SI}/SI	$\sum_{i=1}^{m} H(M_i^t\|M_i^{t-\tau}) - H(X^t\|X^{t-\tau})$	
Mutual Integrated Information[6]	MII / ϕ_I	$I(X^{t-\tau}; X^t) - \sum_{i=1}^{m} I(M_i^{t-\tau}; M_i^t)$	
Modularity [12]	Q	$\frac{1}{m}\sum_{i\neq j}(w_{ij} - \frac{K_i^{in}K_j^{out}}{m})\delta(c_i, c_j)$	
Global Efficiency [9,10]	GE	$\frac{1}{N(N-1)}\sum_{i\neq j}\frac{1}{d(i,j)}$	
Mean Coherence	C	$\frac{2}{N(N-1)}\sum_{i=1}^{N-1}\sum_{j=i+1}^{N}\|r_{ij}\|$	
Mean Connection Strength	μw_{ij}	$\frac{1}{N(N-1)}\sum_{i=1}^{N-1}\sum_{j=i+1}^{N}(w_{ij})$	
Lempel Ziv Complexity [3]	LZs	$\frac{1}{N}\sum_i \frac{LZ(Hb(X_i))}{LZ(Hb(X_i)^{shuffled})}$	Hb is Hilbert transform binarized; 1 if $\mu\|\hat{u}X_i^t\| > \mu\|\hat{u}(X_i)\|$
Amplitude Coalition Entropy [3]	ACE	$\frac{H(vec(Hb(X)))}{H(vec(Hb(X)^{shuffled}))}$	Hb is Hilbert transform binarized; 1 if $\mu\|\hat{u}X_i^t\| > \mu\|\hat{u}(X_i)\|$
Synchrony Coalition Entropy [3]	SCE	$\mu(\frac{SCE_i}{SCE_i(\Psi_t^{i,shuffled})}), SCE_i = -\sum_\psi p(\Psi_t^i)\log p(\Psi_t^i))$	$\Psi_t^i = 1$ if $\psi_{ij}^t < 0.8$ and $\psi_{ij}^t = \|\text{imag}(\hat{u}(X_i^t)) - \text{imag}(\hat{u}(X_j^t))\|$
Directed Transfer Function [13]	DTF	$\sqrt{\frac{\|H_{ij}(f)\|^2}{\sum_{m=1}^{k}\|H_{im}(f)\|^2}}$	

A brief note on notation, but see source articles for details. For PCI^{ST}, T_R denotes nr. of samples in NST_n^{res}, and NST is nr. of threshold ϵ crossings for the principal component n of data in baseline (base) and response (res), with k as a weighting factor. For ϕ^G D_{KL} is the Kullback-Liebler divergence, $\mathcal{M}_G$ is a manifold of probability distributions q for various partitions (with m nr. of parts and the state of each part represented by M_i) of the full system represented by the probability distribution p. The state of the system is represented by X^t at time t, with τ as offset. Generally, H is Shannon entropy, I is mutual information. i/j refers to a given node or channel, $d_{i,j}$ is the distance between i/j (treated as nodes) and is the complement of edge weight $w_{i,j}$, with $k_i = \sum_{i\neq j} w_{i,j}$. r_{ij} is the Pearssons correlation between i/j (treated as channels). For Modularity and Global Efficiency, N is nr. of nodes, m is nr. of edges, and c is community. For DTF, H_{ij} refers to an element of a transfer matrix of a MVAR model, for $i \rightarrow j$ at frequency f. For ACE the matrices are vectorized to $mn \cdot 1$ denoted as $vec(A)$. For LZs and ACE, $Hb^{shuffled}$ means that each binarized channel $Hb(X_i)$ is shuffled to produce a random signal, while for SCE it's the coalition series Ψ_t^i. Note that for the measures GE, Q, and μw_{ij}, these were applied to the DTF based results of the recorded EEG data, the generated data of an auto-regressive model (prefix Est.), and the underlying matrix of the auto-regressive model (prefix Mod.). C was however based on the real (C) and generated data (Est. C), and the noise factor of the auto-regressive model (Mod. C). Finally, note that LZ is based on a compression algorithm that approximates Kolmogorov complexity, and that ϕ^G requires further assumptions to produce a closed form expression (e.g. binary or gaussian distribution).
