# Supplementary figures and images for: Exploring effects of anesthesia on complexity, differentiation, and integrated information in rat EEG
Source: Neurosci Conscious. 2024 May 16;2024(1):niae021. doi: 10.1093/nc/niae021 (PMC11097907; doi:10.1093/nc/niae021)

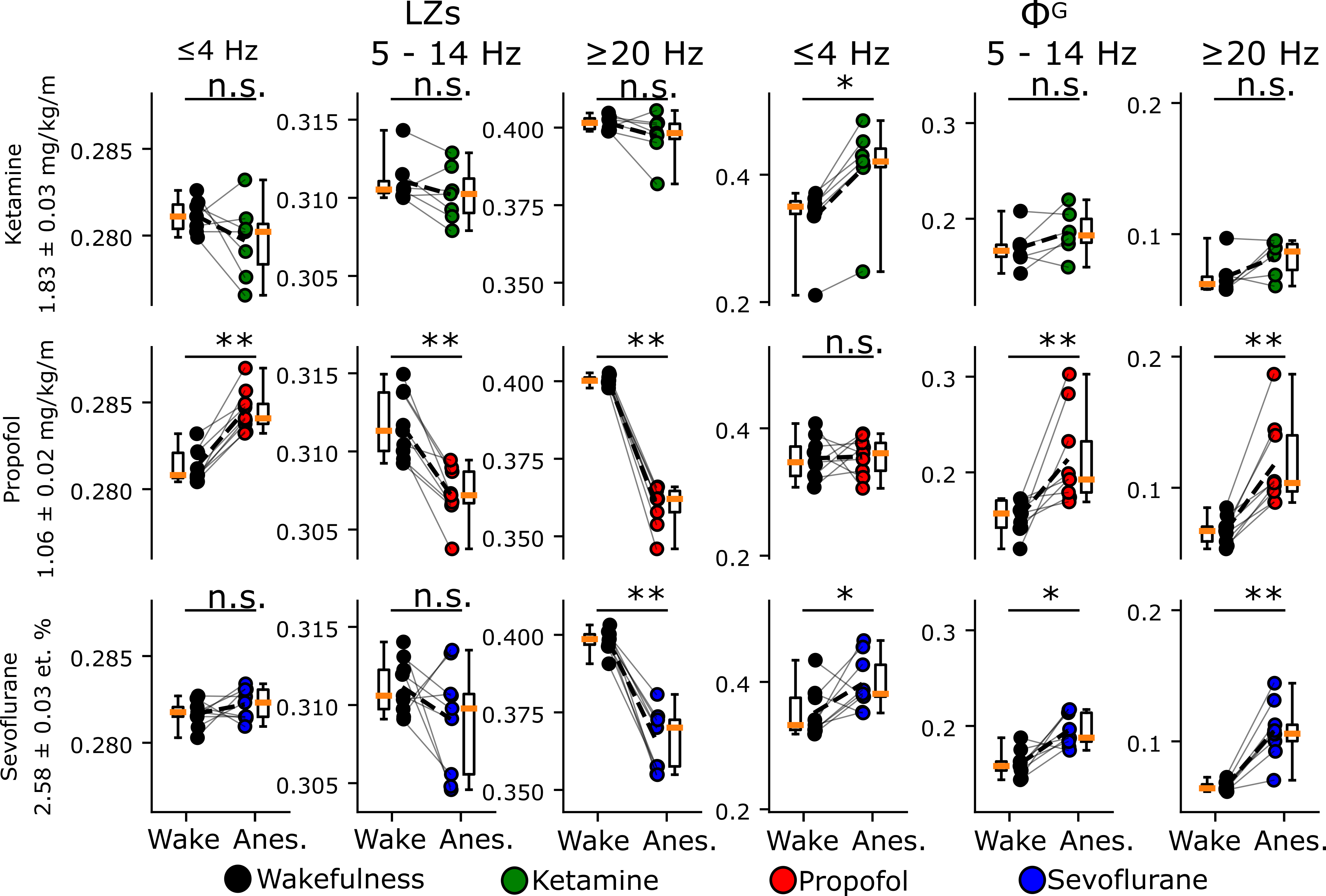

Supplement: niae021_Supp [file niae021_supp.zip › suppl_data/FIG_1A_rev3.png]
